# Reproduction of Gum Tissue

**Published:** 1898-07

**Authors:** George T. Carpenter

**Affiliations:** Chicago.


ARTICLE V.

### REPRODUCTION OF GUM TISSUE.

GEORGE T. CARPENTER, M. D., D. D. S., CHICAGO.

Read before Chicago Dental Society, October, 1897.

What can be done for receding gums? This is answered very quickly by a great many operators, that we can do little to restore this lost tissue; and in my judgment the trouble has been that very little experimental work has been done in this direction, and the literature is nearly silent on the subject. A review of seventeen years shows three articles on sponge grafting and one on the use of resorcin in cases of receded gums.

It is not my purpose in this paper to give the etiology of receding gums, but I would suggest that the receding process be checked and the gum tissue returned to a condition of health before any attempt at reproduction be commenced. All dental operators have seen large proximal and buccal cavities of decay filled with gum tissue, sometimes very vascular, but in other cases this tissue will be found firm and healthy. Ill-fitting plates will sometimes cause a growth of gum tissue. Deposits on the necks of teeth just under the gum margin will in rare cases cause an extra gum development. A lady who was waiting for a friend in my office, called my attention to a full upper plate which she wore. The plate was of rubber and was broken in halves between the central incisors, and in this condition had been worn with comfort for over two years. The gum and mucous membrane of the palate presented a ridge, a section of which would make an inverted T., which extended from the centrals to a line near the soft palate. The membrane was in a fairly healthy condition. The condition of things formed a basis for some experiments. I found that the sharp irregular edges of the plate caused the irritation, and the overhanging walls acted as a protection for the new growth. (I do not tell this as new.)

After some thought and work along this line, I was convinced that if the same conditions could be brought about where gum tissue was deficient, that the tissue would be reproduced; but how to do it was the query. Irritation to cause granulations and protection to prevent their displacement or destruction. Chemical irritants of various kinds were used and their action on the tissue closely watched. Mechanical irritation was also tried, among which was the lancet and the use of barbed or roughened wire, also sharp or irregular edges of different metals inserted in various ways, producing either internal or external irritation of the gums? The manner of protecting the gums was also a study. Several different varnishes were used, also chemicals that formed combinations with the surface of the tissue, producing a film as a protection for the deeper-seated parts. I also used mechanical devices of different forms and materials, many of which were found worthless.

In order to be successful much depends upon the physical and mental condition of the patient, who must thoroughly appreciate the need of the tissues to be reproduced and be willing to co-operate with the dentist and carry out in detail all instructions. Protection of the parts may be secured by a vulcanite rubber hood, which, if carefully made and adjusted, can be worn with comfort. To make the hood, an impression should be taken of the receded gum with modeling compound, taking first only one side, cool the compound and then take the other side, letting the soft compound extend over the hard, remove, place in position and make a model of plaster. I then restore on the model with plaster or wax the absent gum, building as high or a little higher than the surrounding gum. A sheet of thin wax is then slightly warmed and pressed upon the newly built part, letting the wax extend to the surrounding gum and a little distance upon and between the teeth. This may be flasked, packed and vulcanized. Either the vellum rubber or the ordinary vulcanite rubber can be used, as the case may indicate. Platina plate or wire may be vulcanized in the rubber so as to extend between the teeth, and bent so as to hold the rubber hood in position, or ordinary rubber bands may be vulcanized in the rubber so as to be stretched over the teeth for support, or it can be kept in position by silk or wire ligature. Rubber made in this way will form an excellent protection to the parts to be operated on. Tincture of iodin should be used upon the gums until a firm rounded margin is obtained. The irritation is produced just under the gum margin by a clamp made of thin clasp metal, armed with a sharp edge standing at right angles and made of the length and shape to produce the irritation where required. This instrument will hug the root tightly and will keep in position. After adjusting the clamp, the parts are then stimulated with a solution of nitrate of silver ʒi to distilled water ℥i. The rubber hood should now be placed in position and the condition watched from time to time. Granulations can be observed and the growth of gum tissue noted. This is my method of reproducing gum tissue, but I do not claim the results will be the same in all cases. You will find cases where the gum could be extended to the grinding surface or cutting edge of the teeth.

There is another condition which I would call atrophy of the gums. The gums are pale and very thin at their margins, there is an absence of the alveolus, and in some cases V-shaped exposures of the root almost to the apex. Treatment: After all deposits are removed the gums are painted lightly with tincture of iodin every second day, also stimulated at intervals with a solution of nitrate of silver, until the festoon becomes rounded and healthy. In case of the V-shaped exposure, a hood should be made as described above, and after the parts are carefully cleansed antiseptically, the margins should be carefully pared and an incision made on either side, passing down close to the periosteum so as to make two flaps which can be brought together with a stitch, using a very fine needle and a silk ligature. When the edges are made to approximate, flood the parts with nitrate of silver ʒss to the ℥i of distilled water and place the rubber hood in position. Watch carefully for a few days and remove silk as soon as tissues unite. If there is any tendency to gap, touch the edge with crystal nitrate of silver.—*Dental Review.*

